# Association Between Sebum, Total Cholesterol, and Low-Density Lipoprotein (LDL) Cholesterol Levels With Post-acne Keloids

**DOI:** 10.7759/cureus.43096

**Published:** 2023-08-07

**Authors:** Yuli Kurniawati, M. Soleh Rodian, Fifa Argentina, Gita Dwi Prasasty, Dalilah Dalilah, Amanda Nathania

**Affiliations:** 1 Dermatology and Venereology, Dr. Mohammad Hoesin General Hospital, Sriwijaya University, Palembang, IDN; 2 Medical School, Faculty of Medicine, Sriwijaya University, Palembang, IDN; 3 Faculty of Medicine, Sriwijaya University, Palembang, IDN; 4 Dermatology and Venereology Department, Faculty of Medicine, Sriwijaya University, Palembang, IDN

**Keywords:** low-density lipoprotein, acne vulgaris, cholesterol, sebum, post-acne keloid

## Abstract

Background

Prolonged acne inflammation causes scar formation, one of which is post-acne keloids. Sebum, total cholesterol, and low-density lipoprotein (LDL) level can influence post-acne keloids. This study aims to determine the association between sebum, total cholesterol, and LDL levels with post-acne keloids to better define the predisposing factors for this condition.

Methods

This study used primary data involving sociodemographics, clinical features, keloid classification, sebum levels, total cholesterol levels, and LDL levels in post-acne keloid patients at the Dermatology, Venereology, and Aesthetics Outpatient Clinics of Dr. Mohammad Hoesin General Hospital Palembang, Indonesia. Study samples were patients who fulfilled the inclusion and exclusion criteria by consecutive sampling. The data then underwent univariate and bivariate analyses to show the association between variables.

Result

A total of 22 patients with post-acne keloids participated. The subjects presented mostly with major keloids based on the classification (59.1%). The patients were predominantly 21-30 years old (50%) and male (90.9%). The keloids had onsets >six months to one year (45.5%), durations of one to five years (77.3%), and multiple presentations (68.2%). Vancouver Scar Scale (VSS) assessment showed mainly red vascularity (40.9%), mixed pigmentation (68.2%), >5 mm keloid height (59.1%), and firm pliability (40.9%). Most patients presented with pruritus (86.4%) but without pain (54.5%). Most had low levels of sebum (50%), normal total cholesterol (90.9%), and near-optimal LDL level (40.9%). There were no significant association between sebum (p = 1.000), total cholesterol (p = 1.000), and LDL (p = 0.376) levels with post-acne keloids. However, LDL levels above normal were most found in this study (68.2%).

Conclusions

There is no association between sebum, total cholesterol, and LDL levels with post-acne keloids. Despite the fact that LDL level was not statistically significant, there has been a rise in LDL level in the research subjects. Further research with a larger number of subjects and consideration of multicenter study through retrospective/prospective methods and complete lipid profile examinations is still required to provide a more representative study.

## Introduction

Acne is a chronic inflammation skin disorder of the pilosebaceous hair follicles caused by abnormal keratinization of sebaceous gland ducts, increased sebum production due to sebaceous gland enlargement, colonization and proliferation of sebaceous gland ducts by *Propionibacterium acnes* (*P. acnes*), and inflammatory responses arising from the immunological activities of *P. acnes*. Acne can cause hyperpigmentation and scars [1,2]. Acne scars are caused by abnormalities in wound healing processes or post-traumatic resolution of sebaceous gland follicles. Among the types of acne scars are keloids [3]. A keloid is a skin disorder caused by abnormalities in the wound healing process, specifically due to an imbalance of collagen production and degradation creating scar tissues that exceed the wound border. Clinical features of keloids may present as pink, purplish, or hyperpigmented nodules or hard plaques [4]. Keloids vary in size, from a pinhead to an orange with a smooth and glossy overlying epidermis, and may be followed by painful and itchy sensations. Keloids involve certain predilection sites, such as the ears, face, neck, shoulders, chest, upper back, upper extremities, hand palms, and soles of the feet [5-7]. A study on the demographics and clinical characteristics of keloids in Sub-Saharan Africa stated that acne was the second largest cause (20.1%) after trauma (27%) in keloid patients studied [8].

Keloids can be influenced by several factors, which can be divided into systemic and local factors [9], such as ethnicity, genetics, gender, age, trauma, inflammation, and topography. One of the topographic factors of keloids is sebaceous gland density. Sebaceous gland activity is triggered by the androgen dihydrotestosterone hormone. It binds to the androgen receptor in sebaceous glands to induce cell proliferation, differentiation, and lipid regulation [10]. Increased cholesterol level leads to increased androgen hormone level. Low-density lipoprotein (LDL) carries cholesterol to cells and interacts with receptors on the cells to sebaceous glands for sebum synthesis [11,12]. Sebum can interact with T cells in traumatized tissue and cause an inflammatory reaction [13]. A prolonged inflammatory response leads to granulation and tissue repair, initiating scar tissue formation and fibrosis process. It causes the formation of post-acne keloids [14].

It is essential to pay attention to levels of sebum, total cholesterol, and LDL in acne patients toward post-acne keloid incidence. Until now, there has not been any research conducted examining the association between sebum, total cholesterol, and LDL levels with post-acne keloids. This prompted the researchers of the present study to determine the association between levels of sebum, total cholesterol, and LDL with post-acne keloids in post-acne keloid patients at Dr. Mohammad Hoesin General Hospital Palembang, Indonesia.

## Materials and methods

Study participants

This is a cross-sectional observational analytic study. This study was held in August-November 2022 at the Dermatology, Venereology and Aesthetics Outpatient Clinics at Dr. Mohammad Hoesin General Hospital Palembang, Indonesia. The population was post-acne keloid patients. The sample of this study included patients who fulfilled the inclusion and exclusion criteria through consecutive sampling. Inclusion criteria included patients diagnosed with post-acne keloids by either a dermatovenereologist or dermatology, venereology, and aesthetics resident and were willing to sign the agreement as participants. Exclusion criteria included post-acne keloid patients with inflammatory skin diseases for instance folliculitis, smallpox, herpes zoster, hidradenitis suppurativa, and sarcoidosis.

Parameter analysis

This study invited 22 post-acne keloid participants who underwent history taking, physical examinations, and measurement of sebum, total cholesterol, and LDL cholesterol levels. Diagnosis was made based on history taking and physical examinations. Keloids were then classified into major and minor based on their height, pruritus, and pain. Major keloids are defined as >5-mm-high keloids with pruritic and/or pain symptoms, whereas minor keloids are <5-mm-high keloids with pruritic and/or pain symptoms [15].

Clinical features include the onset, duration, number of keloids, and Vancouver Scar Scale (VSS) that measures pigmentation, vascularity, pliability, and height of the keloid [4]. Sebum level was examined using Sebumeter® (SM 815, Courage + Khazaka (CK) Electronic, Cologne, Germany). The sebum level was defined according to the Sebumeter’s manual book. Sebum levels were then classified to high (>220 μg/cm²), normal (>100-220 μg/cm²), and low (<100 μg/cm²). Total cholesterol and LDL levels were examined by a spectrophotometric method (ADVIA 1800® Clinical Chemistry System, Siemens, Erlangen, Germany). Total cholesterol and LDL levels were classified based on the National Cholesterol Education Program Adult Treatment Panel III (NCEP ATP III) criteria. Normal LDL test results are <100 mg/dL, while ≥100 mg/dl are interpreted as above normal. The total cholesterol is also categorized as normal (<200 mg/dL) and above normal (≥200 mg/dL) [16]. 

Statistical analysis

Variables presented in this study include age, gender, clinical features of keloids (onset, duration, number of keloids, and VSS), keloid classification, sebum level, total cholesterol level, and LDL level. Statistical analysis was conducted using IBM SPSS Statistics for Windows, Version 26 (Released 2019; IBM Corp., Armonk, New York, United States) for univariate and bivariate analyses. Univariate analysis shows the number of participants followed by its percentage. Bivariate analysis determines the association between levels of sebum, total cholesterol, and LDL with post-acne keloids using the chi-square test or alternative chi-square test (Fisher/Kolmogorov Smirnov).

## Results

A total of 22 patients showed that most of the post-acne keloid patients were 21 to 30 years old (50%) and predominantly male (90.9%) (Table 1).

**Table 1:** Sociodemographic distribution of post-acne keloid patients (n = 22)

Sociodemography		N (%)
Age	>50 years	1 (4.5)
	41-50 years	0 (0.0)
	31-40 years	7 (31.8)
	21-30 years	11 (50.0)
	10-20 years	3 (13.6)
Gender	Male	20 (90.9)
	Female	2 (9.1)

This study showed that the most clinical features in post-acne keloid patients are those with an onset of >six months to one year (45.5%), duration of one to five years (77.3%), and multiple numbers (68.2%). The VSS assessment showed that each indicator was dominated by red vascularity (40.9%), mixed pigmentation (68.2%), >5 mm high (59.1%), and firm pliability (40.9%). The symptoms are mostly pruritus (86.4%) and painless (54.5%) (Table 2). 

**Table 2:** Distribution of the clinical features of post-acne keloid (n = 22)

Clinical features		N (%)
Onset	>2 years	3 (13.6)
	1-2 years	9 (40.9)
	>6 month-1 year	10 (45.5)
Duration	>10 years	1 (4.5)
	6-10 years	4 (18.2)
	1-5 years	17 (77.3)
Number of keloids	Multiple	15 (68.2)
	Solitary	7 (31.8)
Vascularity	Purplish	4 (18.2)
	Red	9 (40.9)
	Pink	7 (31.8)
	Normal	2 (9.1)
Pigmentation	Hyperpigmented	3 (13.6)
	Mixed	15 (68.2)
	Hypopigmented	2 (9.1)
	Normal	2 (9.1)
Height	>5 mm	13 (59.1)
	2-5 mm	8 (36.4)
	<2 mm	0 (0.0)
	Flat	1 (4.5)
Pliability	Contracture	0 (0.0)
	Ropes	6 (27.3)
	Firm	9 (40.9)
	Yielding	5 (22.7)
	Supple	1 (4.5)
	Normal	1 (4.5)
Pruritus	Yes	19 (86.4)
	No	3 (13.6)
Pain	Yes	10 (45.5)
	No	12 (54.5)

A low sebum level dominated post-acne keloid patients (50%). There was no significant association of the sebum level in post-acne keloid patients (p = 1.000), as presented in Table 3.

**Table 3:** Association between the sebum level and post-acne keloid *p < 0.05 is significantly different.

Sebum level	Post-acne keloid		Total N (%)	p-value
	Major	Minor		
High	1 (4.5)	1 (4.5)	2 (9.1)	1.000
Normal	5 (22.7)	4 (18.2)	9 (40.9)	
Low	7 (31.8)	4 (18.2)	11 (50)	
Total	13 (59.1)	9 (40.9)	22 (100)	

The majority of total cholesterol levels in the post-acne keloid patients were at normal levels (90.9%), as presented in Table 4. Total cholesterol did not differ significantly in post-acne keloid patients (p = 1.000).

**Table 4:** Association between the total cholesterol level and post-acne keloid *p < 0.05 is significantly different.

Total cholesterol level	Post-acne keloid		Total N (%)	p-value
	Major	Minor		
Above normal	1 (4.5)	1 (4.5)	2 (9.1)	1.000
Normal	12 (54.5)	8 (36.4)	20 (90.9)	
Total	13 (59.1)	9 (40.9)	22 (100)	

Most of the LDL levels in post-acne keloid patients were above normal (68.2%). Based on the bivariate test, there was no significant association between the LDL level and post-acne keloid (p= 0.376), as presented in Table 5. Despite the fact that LDL level was not statistically significant, there has been a rise in LDL level among the research subjects.

**Table 5:** Association of the LDL level with post-acne keloid *p < 0.05 is significantly different.

LDL level	Post-acne keloid		Total N (%)	p-value
	Major	Minor		
Above normal	10 (45.5)	5 (22.7)	15 (68.2)	0.376
Normal	3 (13.6)	4 (18.2)	7 (31.8)	
Total	13 (59.1)	9 (40.9)	22 (100)	

## Discussion

This study showed no significant association between sebum level and post-acne keloid. This result is in accordance with the epidemiology study of Liu et al. that shows that most patients with keloid presented with a normal skin type, with numbers as many as 119 out of 240 subjects (47.4%). However, the association of this study is different from that of Liu et al. as there is a significant association between oily skin type and the incidence of keloids (p = 0.001). It is connected to the fact that the normal skin around the keloid has an identical structure to the keloid. Inflammation plays a role in the formation of keloids. This mechanism is initiated by interleukin-1α (IL-1α) in the sebaceous glands. The mediators cause increased proliferation, abnormal differentiation of keratin cells, and accelerate microbial colonization of the skin. Prolonged inflammatory response leads to granulation and tissue repair [17]. Therefore, it formed scars and fibrosis tissues, resulting in post-acne keloids.

There was no significant association between the total cholesterol level and post-acne keloids in our study. A similar study conducted by Luo et al. showed no significant distinction between total cholesterol levels in keloid patients and controls [18]. It can be caused by vascular endothelial growth factor (VEGF) in keloid tissues that accelerate extracellular matrix (ECM) deposition through angiogenesis. Keloid patients have higher serum VEGF levels than normal-skin patients [17,19]. A meta-analysis study by Dai et al. demonstrates that the administration of VEGF/VEGFR (VEGF receptor) inhibitors had a higher incidence of hypercholesterolemia than controls [20]. This is associated with various VEGF mechanisms that can affect the serum lipid metabolism. The function of VEGF-B consists of transcription regulation of fatty acid transporters in circulating blood and cholesterol level regulation through LDL receptors. The inhibition of VEGF-B in type 2 diabetes patients is affecting insulin sensitivity. The systemic blockade of VEGF-C/VEGFR-3 causes changes in insulin sensitivity, so it can be a treatment target in metabolic syndrome. VEGF-D blockade causes an increase in plasma cholesterol levels in mice [21].

Keloid severity can be affected by IL-37 serum as an anti-inflammatory or immunosuppressant by inhibiting adaptive and innate immunity [17,22]. As mentioned in Khattab and Samir’s research, the IL-37 serum level is negatively related to keloid. The IL-37 level was lower in severe keloid patients than in mild keloid patients [22]. Pursuant to Bautista et al.'s study, patients with hypercholesterolemia had a lower risk of IL-37 polymorphism. There was a decrease in serum cholesterol levels in mice with the IL-37 gene compared to controls as it occurs due to AMP-activated kinase (AMPK) activation in the liver [23]. This underlies the large number of post-acne keloid patients with normal cholesterol levels.

There was no significant association between the LDL level and keloid post-acne. Disruption of tumor necrosis factor alpha (TNFα)-VEGF signal cascade by LDL causes a decrease in the VEGF level [21]. VEGF inhibition causes the AKT serine/threonine kinase (AKT)/mechanistic target of rapamycin (mTOR) pathway mechanism to be inhibited. In consequence, the mTOR inhibitor causes an increase in the serum lipid level [20]. AKT/mTOR functions in regulating signal transduction in cell proliferation, apoptosis, metabolism, and angiogenesis. Xu et al. stated that a high LDL level significantly reduced endothelial proliferation. A high level of LDL can lower VEGFR-2 expression related to cell cycle termination, mainly the angiogenesis effect in wound healing [24,25]. Keloid patients have higher VEGF serum levels [17]. Based on this study, post-acne keloids are common in patients with above-normal LDL levels. Although the LDL level was not statistically significant, there has been an increase in the LDL level among the research subjects.

The results of this study showed that post-acne keloid patients mostly had an onset >six months to one year. Once the skin is injured, it will likely take three to 12 months to see the scar appears. After it begins, a keloid tends to grow slowly for months or years. The first sign is usually a thickening of the skin [26,27]. The study results also showed that post-acne keloid patients were mostly pruritic. The itching sensation is due to the presence of neurotransmitters in the lesions. These neurotransmitters consist of histamine, acetylcholine, bradykinin, and proteinase. The itching sensation in the wound healing process is caused by an inflammatory response and partly due to the presence of mast cells in the wound area [28-30].

The limitation of this study was the limited number of participants. Further research with a larger number of participants and multicenter studies through the retrospective/prospective method and complete lipid profile examination are still required to provide more representative study results.

## Conclusions

In this study, there is no significant association between sebum, total cholesterol, and LDL levels with post-acne keloids. Despite the fact that the LDL level was not statistically significant, there has been a rise in the LDL level in the research subjects. Other factors, such as genetic, age, skin tension, habit, and nutrition, that have not been studied should be considered in future research as they might predispose to post-acne keloids. Further research with a larger number of subjects and considering a multicenter study through the retrospective/prospective method and complete lipid profile examination is still required to provide a more representative study.
